# Adapting Gel Wax into an Ultrasound-Guided Pericardiocentesis Model at Low Cost

**DOI:** 10.5811/westjem.2016.10.31506

**Published:** 2016-11-21

**Authors:** Robert Daly, Jason H. Planas, Mary Ann Edens

**Affiliations:** * University of Florida Health Shands Hospital, Department of Emergency Medicine, Gainesville, Florida; † LSU-HSC Shreveport, Emergency Medicine Department, Shreveport, Louisiana

## Abstract

Cardiac tamponade is a life-threatening emergency for which pericardiocentesis may be required. Real-time bedside ultrasound has obviated the need for routine blind procedures in cardiac arrest, and the number of pericardiocenteses being performed has declined. Despite this fact, pericardiocentesis remains an essential skill in emergency medicine. While commercially available training models exist, cost, durability, and lack of anatomical landmarks limit their usefulness. We sought to create a pericardiocentesis model that is realistic, simple to build, reusable, and cost efficient. We constructed the model using a red dye-filled ping pong ball (simulating the right ventricle) and a 250cc normal saline bag (simulating the effusion) encased in an artificial rib cage and held in place by gel wax. The inner saline bag was connected to a 1L saline bag outside of the main assembly to act as a fluid reservoir for repeat uses. The entire construction process takes approximately 16–20 hours, most of which is attributed to cooling of the gel wax. Actual construction time is approximately four hours at a cost of less than $200. The model was introduced to emergency medicine residents and medical students during a procedure simulation lab and compared to a model previously described by dell’Orto.^1^ The learners performed ultrasound-guided pericardiocentesis using both models. Learners who completed a survey comparing realism of the two models felt our model was more realistic than the previously described model. On a scale of 1–9, with 9 being very realistic, the previous model was rated a 4.5. Our model was rated a 7.8. There was also a marked improvement in the perceived recognition of the pericardium, the heart, and the pericardial sac. Additionally, 100% of the students were successful at performing the procedure using our model. In simulation, our model provided both palpable and ultrasound landmarks and held up to several months of repeated use. It was less expensive than commercial models ($200 vs up to $16,500) while being more realistic in simulation than other described “do-it-yourself models.” This model can be easily replicated to teach the necessary skill of pericardiocentesis.

## BACKGROUND

Cardiac tamponade is a life-threatening emergency in which pericardiocentesis may be required. Real-time bedside ultrasound (US) has obviated the need for routine blind procedures in cardiac arrest, and the number of pericardiocenteses being performed has declined. Despite this fact, pericardiocentesis remains an essential skill in emergency medicine that can be performed with a high degree of success.^2^ While commercially available training models exist, cost,^3^ durability, and lack of anatomical landmarks limit their usefulness. Cheaper, do-it-yourself (DIY) models have been described in the literature. Dell’Orto described one in 2013,^1^ in which a tennis ball was placed in a fluid-filled balloon, set on a layer of gel wax in a square container, and then submersed in US gel. This model was easy to build but lacked realism, durability, and cleanliness during use.

## OBJECTIVE

Although low cost, simple, and reusable DIY models have been described,^1^ we sought to create a model that retains those qualities while being more realistic.

## CURRICULAR DESIGN

The model used a red dye-filled ping pong ball (simulating the right ventricle) and a 250cc normal saline (NS) bag (simulating the effusion) encased in an artificial rib cage, held in place by a gel wax/flour solution. The inner saline bag was connected to a 1L saline bag outside of the main assembly to act as a fluid reservoir for repeat uses. The model was mounted loosely to a piece of plywood and covered with TheraBand^TM^ latex exercise resistance bands, which proved to be an excellent skin analog. The materials listed make the cost of the assembly <$200 (Axial skeleton from Amazon $85, gel wax $35, molding bucket $8, ping pong ball $1, NS bag $6, infusion tubing $14, stopcock $3, cyanoacrylate glue $3, flour $2, TheraBand $30; total ~$195). Complete instructions for construction are outlined below.

### Construction

**Figure 1:**
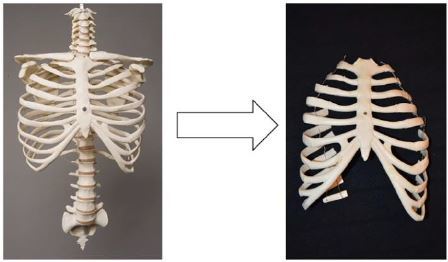
Prepping an artificial rib cage for the first step in creating an inexpensive simulation alternative for teaching pericardiocentesis.

**Figure 2:**
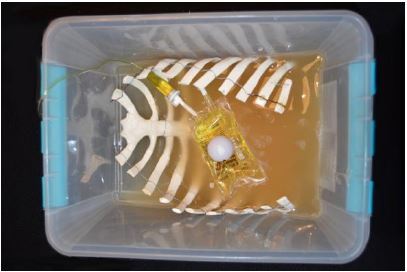
Melted gel wax is used to fill the artificial sternum, covering half of a ping pong ball that simulates the right ventricle.

**Figure 3:**
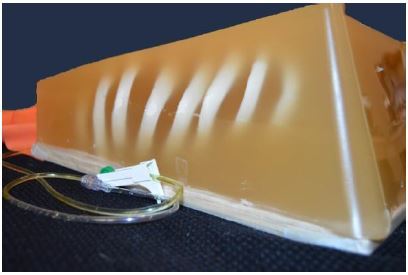
The mold for pericardiocentesis simulation model.

**Figure 4:**
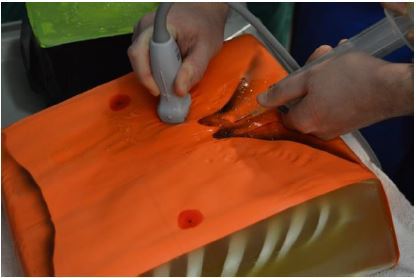
Adding finishing touches to torso model, including a TheraBand skin analog with drawn-on nipples.

1. Prep the artificial rib cage. Remove the posterior portion of the rib cage at the mid-axillary line. This can be done with heavy-duty scissors or trauma shears as seen in Figure 1. The prepped rib cage was then placed into a 20 ×14 × 8 inch container, sternum down.
2. Prepare the internal plumbing.
  - Fill a ping pong ball with red-dyed water via 18g needle. A small second hole next to the injection hole will allow air to escape. Once the ball is as fluid filled and air free as possible, seal the hole with cyanoacrylate glue.
  - Spike a 250cc bag of saline with accessory IV tubing. Inject the bag with 1cc food coloring and mix. Attach a three-way stopcock to the tubing and flush the tubing of air. Manipulate the 250cc bag when flushing the line to ensure that as much air is removed from the bag as possible.
3. Prepare the gel-wax solution. Melt one gallon of gel wax (available at most craft stores) over low-medium heat. Once melted, mix in three tablespoons of flour until dissolved. The flour should be added very slowly to avoid boil-over. Once dissolved, strain away surface foam and discard.
4. Pour the wax to create the mold. This is done in three steps.
  - The first pour: Slowly pour the melted wax solution over the sternum down the rib cage. Fill the container until the wax is just high enough to cover the exposed sternum. Allow mold to cool for 20 minutes.
  - The second pour: First place the 250cc bag on the model in the anatomical location of the anterior portion of the pericardial sac. The IV tubing was allowed to drape over the cooled wax and extend out to leave the area of the container. The ping pong ball was then placed on the 250cc bag in the position of the right ventricle. The wax mixture was re-heated and returned to liquid form. A wooden spoon or stick was used to apply slight pressure to the ping pong ball to hold it in place and maintain safety while the mixture was poured to cover ~1/2 of the ping-pong ball. Now allow mold to cool for 20 min (Figure 2).
  - The third pour: Re-heat the wax solution again, and then pour over the mold until the ribs are submerged. Allow to cool for one hour.
5. Model completion. Invert the container holding the model and remove the container, leaving just the mold as seen (Figure 3). Mount this on plywood, apply a small layer of US gel to the mold, and then cover with the TheraBand skin analog. Markers can then be used to draw nipples and the costal borders (Figure 4).

The dell’Orto model was also constructed per the directions outline in their paper.^1^. Twenty-three learners, comprised of 20 EM residents and three medical students, used and rated both models with a four-question survey. The questions rated from 1 (not well) to 9 (very well) the realism of the models, as well as the ease of recognition of the pericardium, heart, and pericardial effusion.

## IMPACT/EFFECTIVENESS

**Figure 5:**
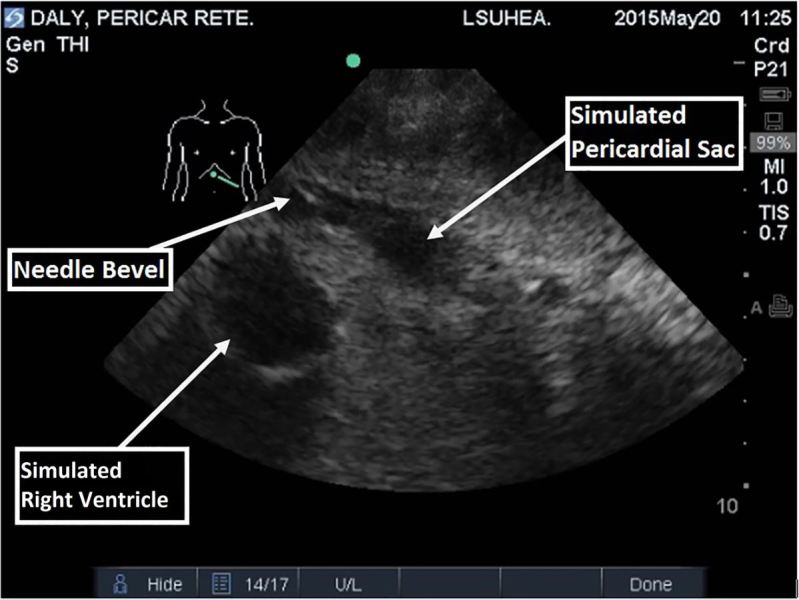
Residents performed ultrasound-guided pericardiocentesis using a model constructed of inexpensive materials.

The model was introduced to EM residents (n=20) and medical students (n=3) during a procedure simulation lab and compared to a model previously described by dell’Orto. The learners performed US-guided pericardiocentesis using both models (US demonstration of our model seen in Figure 5). Learners were given a survey comparing realism of the two models and rated ours 7.8/9 vs 4.5/9 for the previously described model. The survey also showed perceived improvement in the recognition of important structures: pericardium (5.7/9 to 8/9), the heart (5.8/9 to 8.1/9), and the pericardial sac (6.2/9 to 8.4/9). The model performed well for repeated uses over one year. Once the model begins to lose functionality due to multiple needle punctures through the wax and internal plumbing, the wax can be pulled off and re-melted. This limits subsequent reproduction costs to just the replacement of the internal plumbing.

## LIMITATIONS

This model was tested with a small number of residents and medical students, limiting statistical power for results.

## CONCLUSION

In simulation, this model provided both palpable and ultrasound landmarks and held up to several months of repeated use. It was less expensive than commercial models ($200 vs $16,500) while being more realistic in simulation than other described “DIY” models. This model can be replicated to teach the necessary skill of pericardiocentesis.
